# Morphometric study and sexual dimorphism analyses in an Iranian population of *Scorpio maurus* (Arachnida: Scorpionidae)

**DOI:** 10.1002/ece3.8211

**Published:** 2021-10-24

**Authors:** Parisa Soltan‐Alinejad, Saman Parsaei, Ali Dianat, Mahmood Nikbakhtazadeh, Kourosh Azizi

**Affiliations:** ^1^ Department of Medical Entomology and Vector Control School of Health Shiraz University of Medical Sciences Shiraz Iran; ^2^ Department of Health Sciences and Human Ecology California State University San Bernardino California USA; ^3^ Department of Medical Entomology and Vector Control Research Center for Health Sciences Institute of Health School of Health Shiraz University of Medical Sciences Shiraz Iran

**Keywords:** Fars, Iran, morphometric, *Scorpio maurus*, Scorpionidae, sexual dimorphism

## Abstract

Natural selection and sexual selection are cardinal factors in shaping the body of animals such as scorpions. *Scorpio maurus* (Scorpiones: Scorpionidae) has a worldwide distribution. Sexual dimorphism has been reported from this species in a study in Egypt. Morphometry is used to determine the sexual dimorphism between the two sexes. In the current study, scorpions were collected from six locations of the southern and northern provinces of Fars, Iran. In this study, 53 morphological characters of 15 specimens of each sex of *Scorpio maurus* were studied based on statistical analyses; however, dimorphism was only observed in 21 morphological characters, including chelicerae and carapace length, pedipalp characters, width of the second segment of metasoma, telson and pectin length, number of left pectin teeth, and some of the leg's segments. It means that these characters are in the control of sexual and natural selection. This study was performed for the first time on *Scorpio maurus* species in Iran.

## Graphical abstract

*Scorpio maurus* was collected and evaluated for morphometric study and sexual dimorphism analyses for the first time in Iran. 53 morphological characters of 15 specimens of each sex of *S. maurus* were studied. Sexual dimorphism was observed in 21 morphological characters of *S. maurus* based on statistical analyses. Asymmetry was observed in the number of right and left pectin teeth of *S. maurus*.

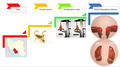

## 1 INTRODUCTION

Natural selection and sexual selection are cardinal factors in shaping the body of animals such as scorpion (Darwin, 1859). Natural selection favors morphologies that meliorate reproduction, growth, survival, and consequent in enhanced competence for a given environment. Sexual selection favors morphologies that comfort the mating process through intra‐ and intersexual competition (Andersson, 1994; Simmons, 2001). Arthropods often display sexual dimorphism which can be limited to certain body parts. Sex differences in body part size have behavioral and, maybe, ecological effects (Miller et al., 2016). One aspect of sexual dimorphism is often the difference in overall body size between the two sexes (Fox et al., 2015).

Members of order Scorpiones have been living on the Earth for more than 400 million years (Ortiz et al., 2015). Many studies have already indicated the sexual dimorphism in different species of scorpions (Carlson et al., 2014). Mature male scorpions often feed less and more in search of females. However, the females move very little because of weight gain during gestation period for several months (Miller et al., 2016; Polis, 1990b; Polis & Farley, 1979). *Scorpio maurus* (Scorpiones: Scorpionidae) has been particularly reported from Middle East, such as Iran (Fet et al., 2000). The venom of this species is a mixture of neurotoxin peptides, which usually does not cause any human mortality (Abdel‐Rahman et al., 2009). So far, no study has been conducted related to the morphometric study and sexual dimorphism analyses of *Scorpio maurus* in Iran, based on statistical analyses. Statistical methods for analyzing the characters scaling are more sensitive for evaluating characters that may still be under the control of sexual or natural selection (Packard & Boardman, 1999). In this study, we analyzed 53 morphological characters between the sexes.

## 2 METHODS

### 2.1 Study area, scorpion collection, and identification

**FIGURE 1:**
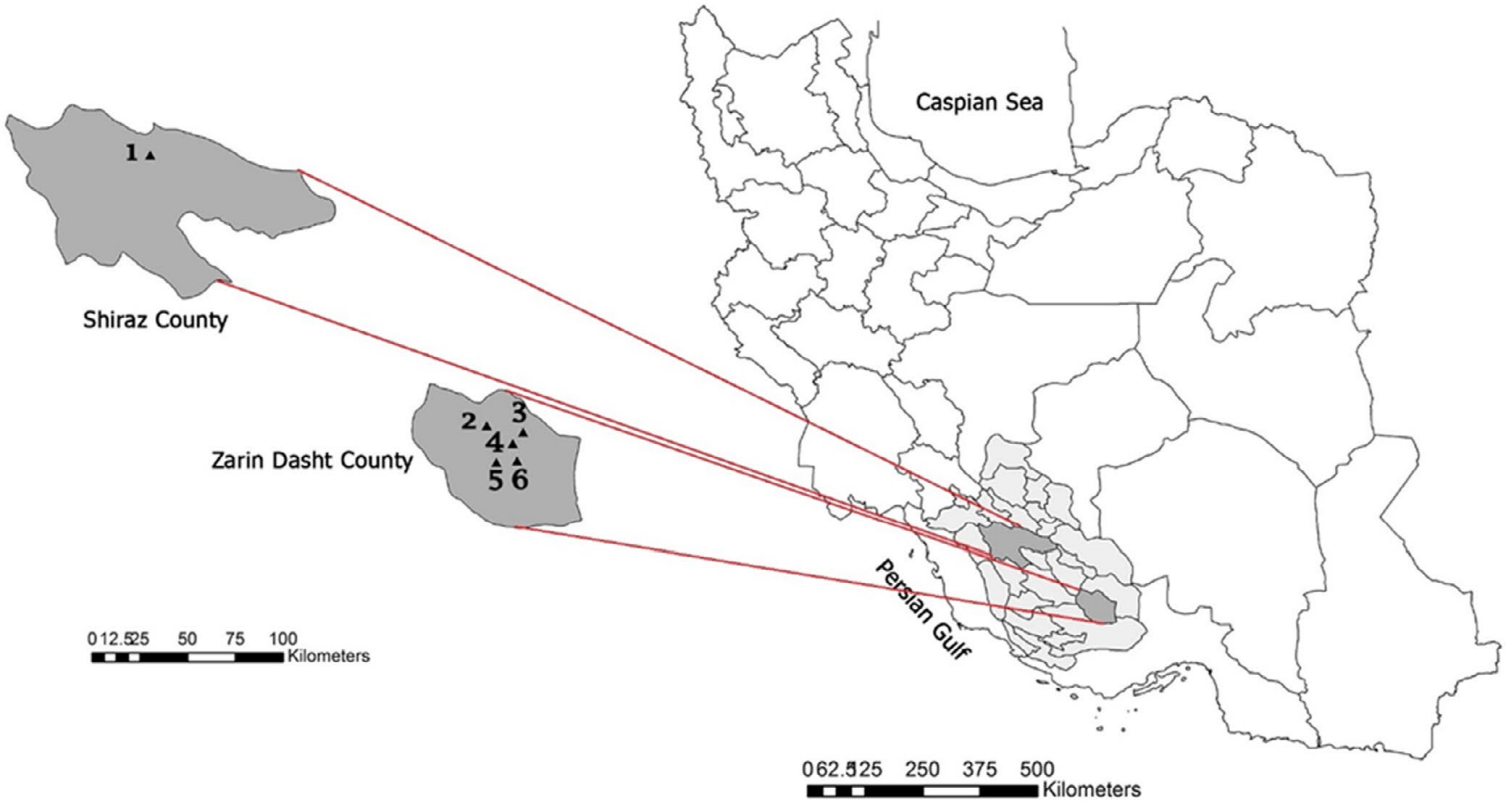
Iran, Fars Province is highlighted in Shiraz County; map shows selected sites in various part of Fars Province for *S*.* maurus* collecting (1) Bajgah, (2) Chah Sabz village, (3) Gaz‐Tavileh village, (4) Hajji Tahereh village, (5) Golkuyeh village, and (6) Chah Zebar village

Scorpions were collected from March to June 2020 in six locations of the southern and northern provinces of Fars. The geographical coordinates of sampling sites (Figure 1) were as follows: Chah Sabz village (28°26′46.6″N 54°24′10.6″E), Hajji Tahereh village (28°21′35.4″N 54°32′23.8″E), Chah Zebar village (28°16′44″N 54°34′3″E), Golkuyeh village (28°59′16.4″N 54°16′56.5″E), Gaz‐Tavileh village (28°15′03.6″N 54°21′49.9″E), and Bajgah (29°43′05.2″N 52°35′16.8″E). Scorpions were collected at daytime by inspecting their potential hiding sites, for example, under rocks, and at night by UV blacklight. We collected a sample of 70 mature and immature scorpions. Mature male specimens were separated from the immatures by the genital papillae. In order to distinguish mature females from immatures, the smallest gravid scorpions were chosen as the index of identification (Abdel‐Nabi et al., 2004). All collected scorpions were preserved in 70% ethanol and transferred to the Medical Entomology Laboratory at Shiraz University of Medical Sciences. Identification to the species level was made by using the key and descriptions of H Barahoei et al study (Barahoei et al., 2020).

### 2.2 Morphometric study

In the current study, 53 morphological characters of 15 males and 15 female specimens of *Scorpio maurus* were measured and analyzed. For checking all six populations, about two mature members (male and females) of each location were chosen as candidates and examined. Inspired by previous study (Abdel‐Nabi et al., 2004), in addition to measuring the characters reported from previous studies, some new factors were examined. The measured morphological characters were as follows: the total body length, length and width of carapace, pedipalp (total length and width, fixed finger length and width and also length of the movable finger), length, width and thickness of the metasomal segments 1–5, length, width and thickness of telson, length and width of pectin, right and left pectinal teeth and chelicerael length. Measurements (in millimeter) were carried out using a precision vernier caliper of with a tolerance of 0.02 millimeter (mm).

### 2.3 Statistical analyses

Shapiro–Wilkes test has been performed to test for the non‐normality of the data. Because the data followed a non‐normal distribution, nonparametric Mann–Whitney *U* test was performed for data analysis with SPSS (version 22).

## 3 RESULT

### 3.1 Total body length and weight

**FIGURE 2:**
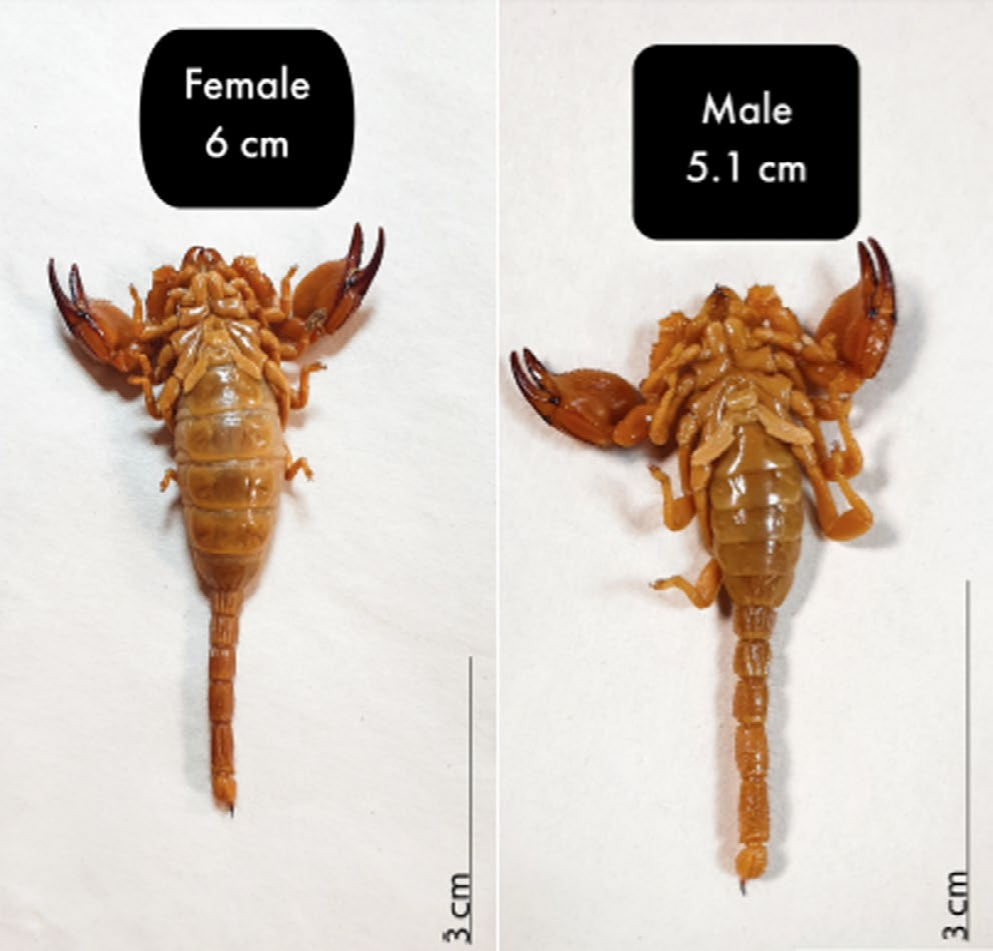
The mean ± SD of total body length of *Scorpio maurus* males (5.2 ± 0.71) and females (5.4 ± 0.28), showing the longer size of females than males

Mean ± SD of the total body length of males and females was 5.20 ± 0.71 mm and 5.40 ± 0.28 mm, respectively. Mean ± SD of the body width of male and female specimens was 1.50 ± 0.20 mm and 1.60 ± 0.31 mm, respectively (Table 1). The *Scorpio maurus* body length is shown in Figure 2.

**TABLE 1:** The mean ± SD of measured morphological characters of body weight, body length, carapace, and chelicerae on *Scorpio maurus*

Morphological traits	Males (Mean ± SD)	Females (Mean ± SD)	*p*‐Value
Body weight (mm)	1.50 ± 0.20	1.60 ± 0.31	.801
Body length (mm)	5.20 ± 0.71	5.40 ± 0.28	.357
Carapace			
Length** (mm)	7.50 ± 0.82	8.00 ± 0.37	.044
Width (mm)	6.50 ± 0.74	6.70 ± 0.31	.772
Chelicerae length** (mm)	4.70 ± 0.62	4.40 ± 0.41	.008

Note

**Shows that difference is significant at the .05 level.

### 3.2 Carapace

According to our analysis, the mean ± SD of carapace length was 7.50 ± 0.82 mm in males and 8.00 ± 0.37 mm in females, while the carapace width was 6.50 ± 0.74 mm in males and 6.70 ± 0.31 mm in females (Table 1).

### 3.3 Chelicerae

**FIGURE 3:**
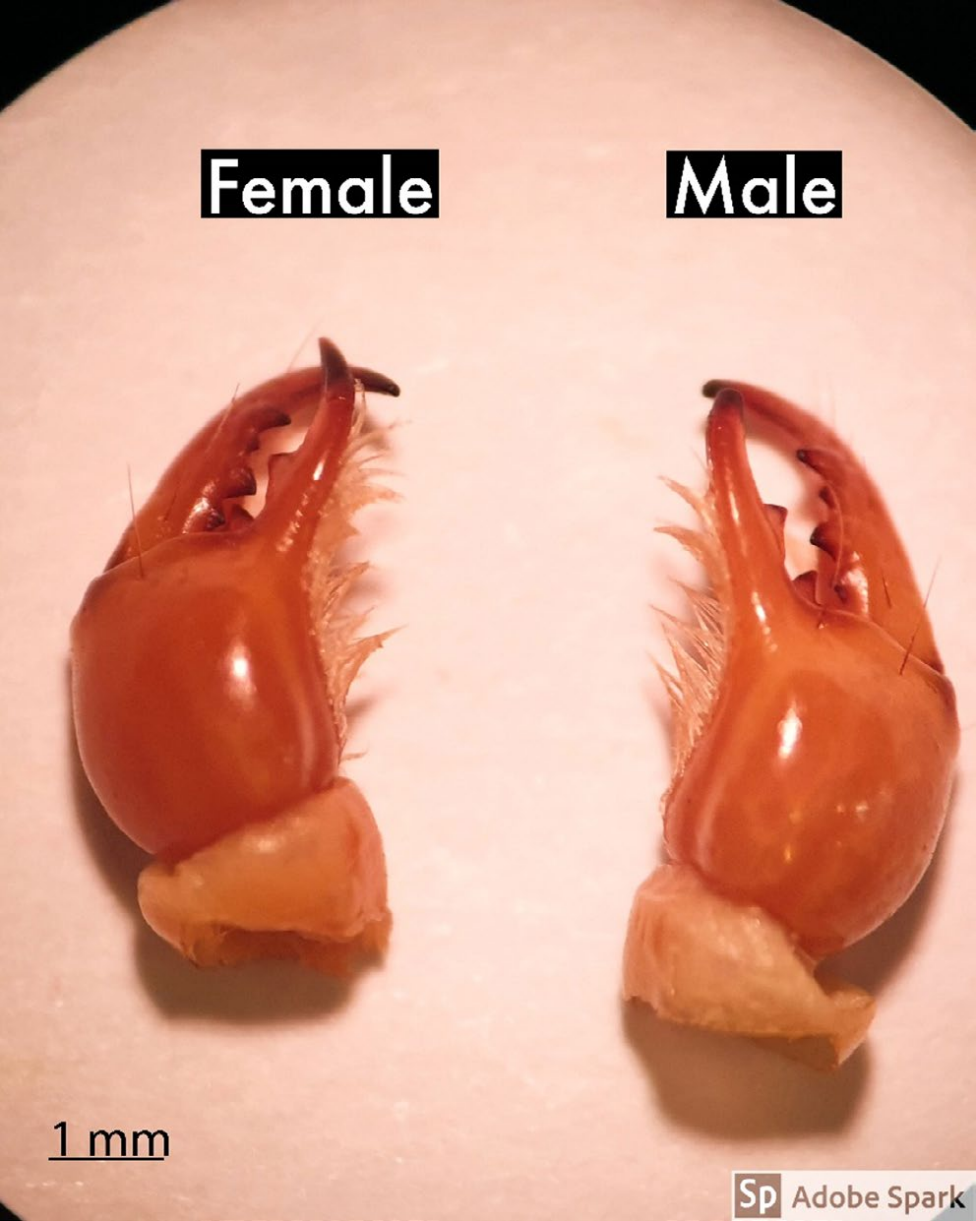
The mean ± SD of the chelicerae total length of *Scorpio maurus* males (4.7 ± 0.62) and females (4.4 ± 0.41), showing the increased length in males

The mean ± SD of chelicerae length was 4.70 ± 0.62 mm in males and 4.40 ± 0.41 mm in females (Table 1). The *Scorpio maurus* chelicerae are shown in Figure 3.

### 3.4 Pedipalp

In this study, five main features of pedipalps were measured as follows: The mean ± SD of pedipalp total length was 21.00 ± 1.70 mm in males and 22.00 ± 1.25 mm in females. Other measured characters about pedipalp in both sexes and the exact *p*‐value of them are shown in Table 2.

**TABLE 2:** The mean ± SD of measured morphological characters of pedipalp on *Scorpio maurus*

Morphological traits		Males (Mean ± SD)	Females (Mean ± SD)	*p*‐Value
Pedipalp				
Total length (mm)		21.00 ± 1.70	22.00 ± 1.25	.282
Movable finger	Length** (mm)	5.70 ± 0.59	7.33 ± 0.40	.000
	Width** (mm)	1.40 ± 0.33	1.90 ± 0.50	.004
Fixed finger	Length** (mm)	10.60 ± 1.05	11.83 ± 0.52	.000
	Width** (mm)	6.20 ± 0.56	6.60 ± 0.48	.047

Note

**Shows that difference is significant at the .05 level.

### 3.5 Pectine

**FIGURE 4:**
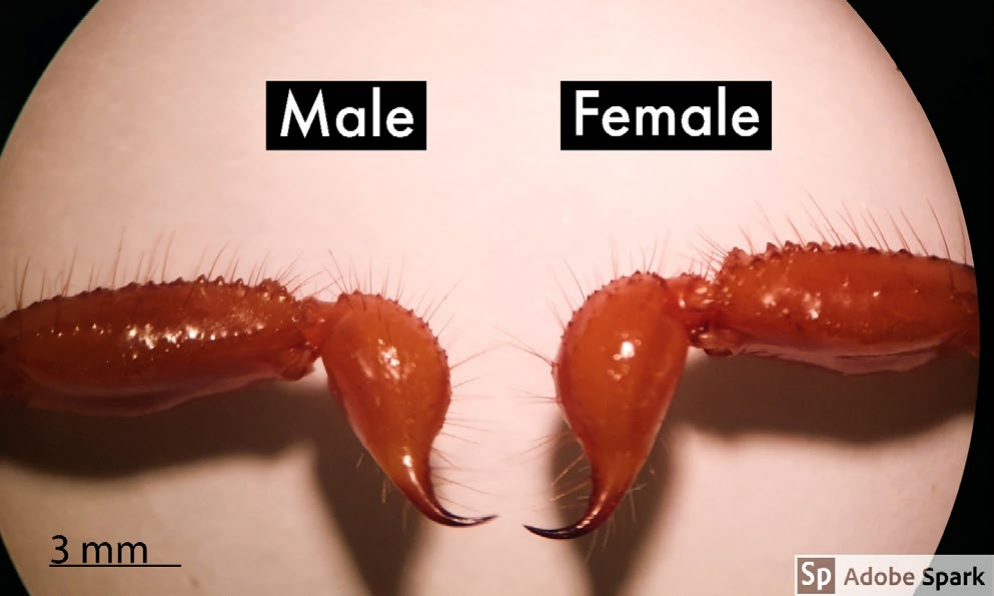
The mean ± SD telson of *Scorpio maurus* males (5.13 ± 0.61) and females (5.6 ± 0.63), showing increased teslon length in females than males

Mean ± SD of pectine length and width in males were 4.73 ± 0.07 mm and 1.06 ± 0.25 mm, whereas the ones of females were 4.5 ± 0.18 mm and 1.00 ± 0.00 mm individually. The measured mean ± SD of the right and left side of pectine teeth were 12.53 ± 0.74, and 12.86 ± 0.35 in males and 12.13 ± 0.74 and 12.0 ± 0.70 in females, respectively (Table 3). Figure 4 shows both sides of pectine in *Scorpio maurus* and the exact *p*‐value.

**TABLE 3:** The mean ± SD of measured morphological characters of pectine on *Scorpio maurus*

Morphological traits		Males (Mean ± SD)	Females (Mean ± SD)	*p*‐Value
Pectine				
Length** (mm)		4.73 ± 0.07	4.50 ± 0.18	.000
Width (mm)		1.06 ± 0.25	1.00 ± 0.00	.317
Teeth number	Right	12.53 ± 0.74	12.13 ± 0.74	.128
	Left**	12.86 ± 0.35	12.00 ± 0.70	.001

Note

**Shows that difference is significant at the .05 level.

### 3.6 Metasoma

Mean ± SD of metasoma length was 18.33 ± 2.08 mm in males and 18.43 ± 0.67 mm in females. In males and females, mean ± SD of the length, width, and thickness of the metasomal segments and the exact *p*‐value of them are shown in Table 4. As indicated in Table 1, the length of each metasomal segment is increased from base to the apex; however, the width and thickness are decreased.

**TABLE 4:** The mean ± SD of measured morphological characters of metasoma on *Scorpio maurus*

Morphological traits		Males (Mean ± SD)	Females (Mean ± SD)	*p*‐Value
Metasoma				
Total length (mm)		18.33 ± 2.08	18.43 ± 0.67	.071
Segment 1	Length (mm)	2.86 ± 0.35	2.83 ± 0.24	.326
	Width (mm)	3.46 ± 0.22	3.53 ± 0.29	.487
	Height (mm)	2.86 ± 0.35	3.00 ± 0.00	.150
Segment 2	Length (mm)	2.86 ± 0.35	3.00 ± 0.00	.150
	Width**(mm)	2.93 ± 0.31	3.23 ± 0.25	.008
	Height (mm)	2.86 ± 0.35	3.00 ± 0.00	.150
Segment 3	Length (mm)	3.40 ± 0.38	3.30 ±.36	.202
	Width (mm)	2.86 ± 0.35	3.00 ± 0.00	.150
	Height (mm)	2.76 ± 0.37	2.96 ± 0.12	.065
Segment 4	Length (mm)	3.93 ± 0.45	3.93 ± 0.17	.774
	Width (mm)	2.86 ± 0.35	3.00 ± 0.00	.150
	Height (mm)	2.36 ± 0.29	2.50 ± 0.00	.074
Segment 5	Length (mm)	5.26 ± 0.62	5.36 ± 0.29	.831
	Width (mm)	2.76 ± 0.37	2.93 ± 0.17	.169
	Height (mm)	2.33 ± 0.24	2.43 ± 0.17	.203

Note

**Shows that difference is significant at the .05 level.

### 3.7 Telson

The mean ± SD of telson length, width, and thickness were 5.13 ± 0.61 mm, 2.76 ± 0.37 mm, and 2.43 ± 0.31 mm in males. The same characters were measured in females as 5.60 ± 0.63 mm, 2.90 ± 0.20 mm, and 2.46 ± 0.12 mm, respectively (Table 5). Telson of both sexes is shown in Figure 4.

**TABLE 5:** The mean ± SD of measured morphological characters of telson on *Scorpio maurus*

Morphological traits	Males (Mean ± SD)	Females (Mean ± SD)	*p*‐Value
Telson			
Length** (mm)	5.13 ± 0.61	5.60 ± 0.63	.017
Width (mm)	2.76 ± 0.37	2.90 ± 0.20	.334
Height (mm)	2.43 ± 0.31	2.46 ± 0.12	.944

Note

**Shows that difference is significant at the .05 level.

### 3.8 Legs

**FIGURE 5:**
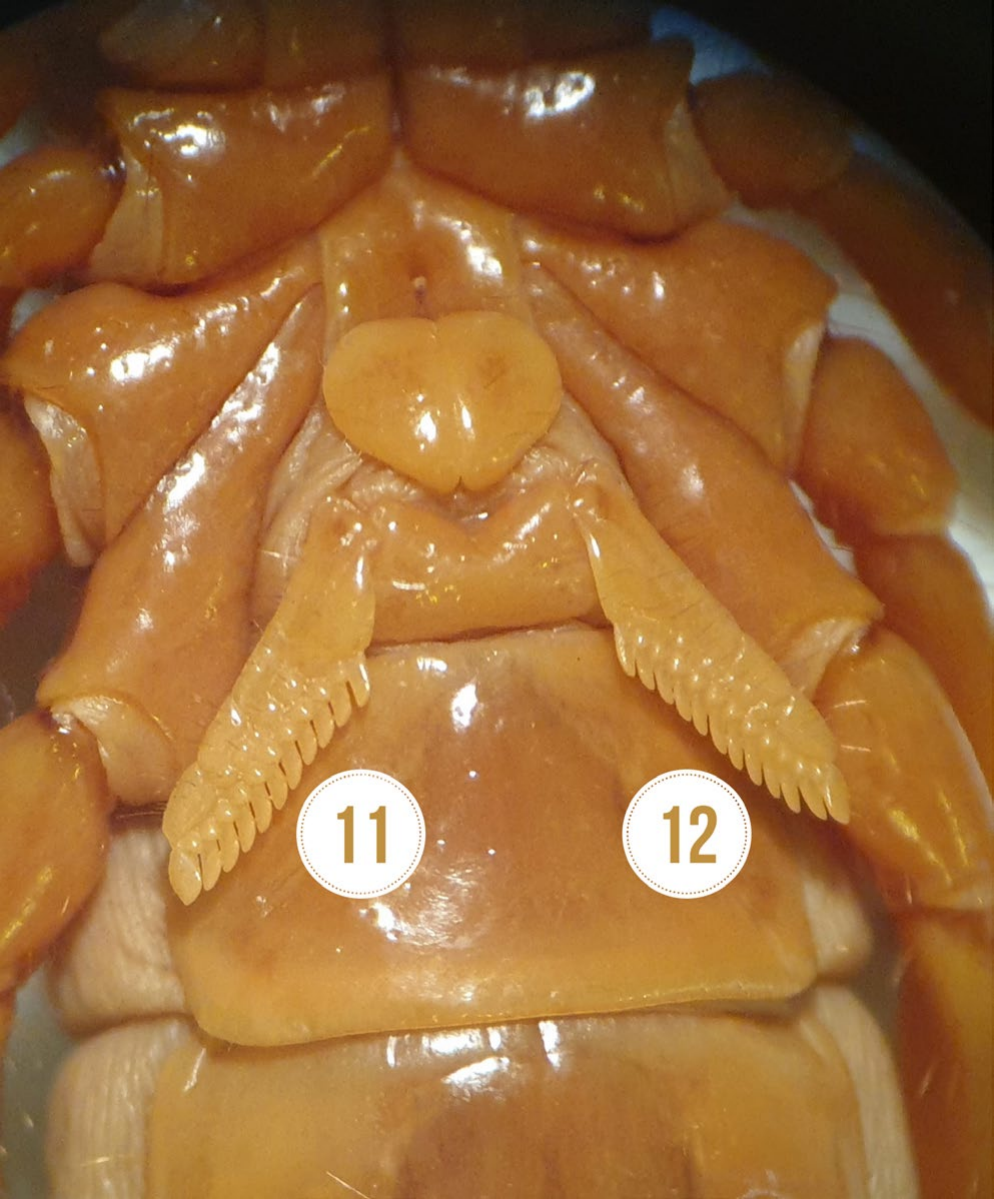
(a) The pectinal teeth of females *Scorpio maurus* showing asymmetry in pectinal teeth on both sides, (b) The legs of *Scorpio maurus*

The mean ± SD of all leg segments (coxa, trochanter, femur, patella, tibia, and tarsus) in both sexes and the exact *p*‐value of them are indicated in Table 6. Legs 1–4 of the *Scorpio maurus* are shown in Figure 5.

**TABLE 6:** The mean ± SD of measured morphological characters of leg on *Scorpio maurus*

Morphological traits		Males (Mean ± SD	Females (Mean ± SD)	*p*‐Value
Leg				
First leg	Coxa (mm)	1.00 ± 0.00	1.00 ± 0.00	1.000
	Trochanter (mm)	1.00 ± 0.00	1.00 ± 0.00	1.000
	Femur (mm)	3.00 ± 0.00	3.00 ± 0.00	1.000
	Patella (mm)	3.00 ± 0.00	3.00 ± 0.00	1.000
	Tibia** (mm)	1.50 ± 0.00	2.00 ± 0.00	.000
	Tarsus** (mm)	2.50 ± 0.00	3.00 ± 0.00	.000
Second leg	Coxa (mm)	3.00 ± 0.00	3.00 ± 0.00	1.000
	Trochanter (mm)	1.00 ± 0.00	1.00 ± 0.00	1.000
	Femur (mm)	4.00 ± 0.00	4.00 ± 0.00	1.000
	Patella (mm)	3.00 ± 0.00	3.00 ± 0.00	1.000
	Tibia** (mm)	1.50 ± 0.00	2.00 ± 0.00	.000
	Tarsus** (mm)	2.50 ± 0.00	3.00 ± 0.00	.000
Third leg	Coxa (mm)	3.00 ± 0.00	3.00 ± 0.00	1.000
	Trochanter (mm)	2.00 ± 0.00	2.00 ± 0.00	1.000
	Femur** (mm)	4.50 ± 0.00	5.00 ± 0.00	.000
	Patella** (mm)	4.50 ± 0.00	5.00 ± 0.00	.000
	Tibia** (mm)	2.00 ± 0.00	3.00 ± 0.00	.000
	Tarsus** (mm)	3.00 ± 0.00	3.50 ± 0.00	.000
Fourth leg	Coxa (mm)	4.00 ± 0.00	4.00 ± 0.00	1.000
	Trochanter (mm)	3.50 ± 0.00	3.50 ± 0.00	1.000
	Femur** (mm)	6.00 ± 0.00	6.50 ± 0.00	.000
	Patella** (mm)	5.00 ± 0.00	5.50 ± 0.00	.000
	Tibia** (mm)	3.00 ± 0.00	3.50 ± 0.00	.000
	Tarsus** (mm)	3.00 ± 0.00	4.00 ± 0.00	.000

Note

**Shows that difference is significant at the .05 level.

### 3.9 Sexual dimorphism analyses

Altogether, analysis of sexual dimorphism included significant differences (*p*‐value <.05) in size of 21 morphological characters of males and females *Scorpio maurus*, as follows: chelicerae length, carapace length, pedipalp characters, width of the second segment of metasoma, telson length, pectine length, number of the left pectine teeth, tibia and tarsus in the first and second leg, and also femur, patella, tibia, and tarsus in the third to the fourth legs.

## 4 DISCUSSION

Morphometric studies and analysis of sexual dimorphism play a very important role in identifying features under the control of sexual and natural selection in animals. Although numerous studies about scorpion dimorphism are performed in Iran, morphometric study and sexual dimorphism analyses in *Scorpio maurus* have never been studied before. Morphometric study on *Scorpio maurus* has been conducted based on 53 morphological characters. Carapace, pedipalp, metasoma, and pectine are the important body parts of scorpions in a morphometric study (Booncham et al., 2007).

### 4.1 Total body length and weight

Similar to the studies on *Mesobuthus eupeus*, the females’ total body length 61.05 ± 2.20 mm was significantly longer than that of males 52.61 ± 3.29 mm (Ebrahimi et al., 2020). Furthermore, the total body length of the females of the Egyptian populations of *Scorpio maurus* was also statistically longer than the males (Abdel‐Nabi et al., 2004). No significant difference (*p*‐value >.05) has found between males’ and females’ weight. Similarly, Dehghani et al. also did not report a significant difference between weight of the two sexes of *Odontobuthus doriae* (Dehghani et al., 2019). All this information is consistent with the results of this study. It is because that the total body length is likely reflects both overall body size and metasoma length. Therefore, the discussion about its sexual dimorphism roles depends on these two factors.

### 4.2 Chelicerae

This character has not been examined in some other morphometric studies on *Mesobuthus eupeus* (Ebrahimi et al., 2020), Egyptian *Scorpio maurus* (Abdel‐Nabi et al., 2004), *Heterometrus laoticus* (Booncham et al., 2007), and *Androctonus crassicauda* (Ozkan et al., 2006). Sexual dimorphism of chelicerae has been studied in the burrowing species of families Scorpionidae, Vaejovidae, Diplocentridae, and Bothriuridae (Polis, 1990a). The measured features of chelicerae depend on the behavior of species. Burrowing scorpions loose soil by chewing action of the chelicerae (Newlands, 1972). Females of the burrowing species make a unique burrow in the soil based on their maternal considerations and parturition (Carrera et al., 2009). During mating, males hold and guide females toward the deposited spermatophore (Peretti, 1993; Polis, 1990a). It seems that the length of the chelicerae in male scorpions plays an important role in controlling the female and therefore can be one of the success factors in mating. Therefore, based on natural selection, it can be concluded that male scorpions with longer chelicerae have higher reproductive competence, and over time, this factor has developed among males and has led to improved survival in the wild.

### 4.3 Carapace

About this feature, the difference was also observed in *Mesobuthus eupeus* (Ebrahimi et al., 2020), Egyptian populations of *Scorpio maurus* (Abdel‐Nabi et al., 2004), *Heterometrus laoticus* (Booncham et al., 2007), and *Androctonus crassicauda* (Ozkan et al., 2006). Females take care of their offspring; so width carapace would be a good point for the survival of their juveniles (Booncham et al., 2007). Therefore, female scorpions with larger carapace have a higher survival rate and sexual selection competence.

### 4.4 Pedipalp

In contrast to a prior study by Abdel‐Nabi et al., who also studied *Scorpio maurus*, no significant difference was found between the two sexes of the Iranian populations of *Scorpio maurus* (*p*‐value >.05) (Abdel‐Nabi et al., 2004). Iranian and Egyptian populations are allopatric populations, which explains why these two are different in the size of pedipalp.

Similar to other studies on *Pseudouroctonus brysoni* (Ayrey & Soleglad, 2020) and *Mesobuthus eupeus* (Ebrahimi et al., 2020), we also found significant differences (*p*‐value <.05) in length of the movable and fixed fingers of pedipalpi of the both sexes of *Scorpio maurus*. A significant difference (*p*‐value <.05) in the width of the movable and fixed fingers of pedipalpi was also observed. In an interesting study on *Hadrurus arizonensis*, it was found that the length of pedipalp in males was longer than that of the females (Fox et al., 2015). Pedipalps have an important role in scorpions’ defense, preying, and mating (Casper, 1985; Fox et al., 2015). Female arachnids, including spiders and scorpions, have a highly predatory activity, and therefore, female pedipalps are bigger than males (29, 30). This is an important natural selection factor for female survives.

### 4.5 Pectine

The pectine length, width, and teeth of both sides were bigger in males than in females. Similar to our observations, Perreti et al. (2001) have also reported these differences in the pectine teeth of *Bothriurus bonariensis* between males and females. Pectine teeth have been reported as 26.93 ± 0.88 in males and 22.20 ± 1.00 in females of *Mesobuthus eupeus* (Ebrahimi et al., 2020). Similarly, the number of pectin teeth was higher in males of *Hottentotta zagrosensis*, *Odontobuthus doriae*, and *Hadrurus arizonensis* than in females (Dehghani et al., 2019; Fox et al., 2015; Tandis et al., 2017). There are some sensory organs in scorpions’ pectine which can detect physical and chemical features of the substrate (Gaffin & Brownell, 1997; Kladt et al., 2007; Steinmetz et al., 2004). Males need the pectines’ sensory organ to deposit the spermatophore in an appropriate substrate (Gaffin & Brownell, 1992; Melville et al., 2003) and also to follow the females’ pheromones in order to find their exact niche (Jiao & Zhu, 2009; Melville, 2000). Features of pectines appear to be similar in a wide range of scorpion families including Buthidae, Bothriuridae, Scorpionidae, and Caraboctonidae.

The number of pectine teeth on both sides of the body was estimated in this study. Similar to our result, those differences in pectine teeth have been also observed in other studies such as the one on the Egyptian populations of *Scorpio maurus* (Abdel‐Nabi et al., 2004) and *Heterometrus laoticus* (Booncham et al., 2007). These differences may indicate an asymmetry in scorpions; however, further studies are required to verify such differences.

### 4.6 Metasoma

The length of female's metasoma in *Scorpio maurus* was longer than male's one, but no significant difference was observed (*p*‐value >.05). It is similar to previous studies about the length of metasoma in Egyptian populations of *Scorpio maurus* (19) and *Mesobuthus eupeus* (Ebrahimi et al., 2020). In contrast, the length of metasoma was longer in the male specimens of *Hadrurus arizonensis* (Fox et al., 2015). Similar to the study on the Egyptian *Scorpio maurus* (Abdel‐Nabi et al., 2004), the second segment indicated a significant difference between males and females. The third segment of metasoma also indicated a significant difference between the two sexes of the Egyptian populations. These differences might be attributed to different geographical populations (Abdel‐Nabi et al., 2004). Like Carlson et al. study on *Centruroides vittatus*, our measurements also confirmed that the width of metasomal segments was narrower in males compared to those of females (Carlson et al., 2014). According to previous studies, narrower metasoma in males could help them reduce their total weight and therefore accelerate their moving during defending, mating, sexual sting, or preying behaviors (Oufiero & Garland, 2007), which can be the key factors of natural selection in enhancing the growth, survival, and reproduction in environment.

### 4.7 Telson

Like the Egyptian *Scorpio maurus* (Abdel‐Nabi et al., 2004) and *Mesobuthus eupeus* (Ebrahimi et al., 2020), the telson length of female *Scorpio maurus* was significantly larger than males in our studies (*p*‐value <.05). The telson was however longer in males than females of *Heterometrus laoticus* (Booncham et al., 2007) and of equal size in *Hadrurus arizonensis* (Fox et al., 2015). This sexual dimorphism in this species might reflect intersexual differences in venom use.

### 4.8 Legs

To find any difference between the leg sizes of the two sexes, all leg segments were measured. A significant difference in some leg segments is herein reported for the first time (*p*‐value <.05). The femur length was increased from the first to the fourth leg. On the other hand, the length of patella, tibia, and tarsus was increased from the second to the fourth leg in both sexes. Due to the connection of the legs to prosoma, the results prove these conditions are necessary to maintain the body balance in scorpions.

The results of current study show that the sexual dimorphism was only observed in 21 morphological characters, including chelicerae and carapace length, pedipalp characters, width of the second segment of metasoma, telson and pectin length, number of left pectin teeth, and some of the leg's segments. It means that these characters are in the control of sexual and natural selection.

## CONFLICT OF INTEREST

The authors declare no conflicts of interest.

## AUTHOR CONTRIBUTION

**Parisa Soltan‐Alinejad:** Conceptualization (equal); Data curation (equal); Formal analysis (equal); Resources (equal); Visualization (equal); Writing‐original draft (equal); Writing‐review & editing (equal). **Saman Parsaei1:** Data curation (equal); Investigation (equal). **Ali Dianat:** Data curation (equal); Investigation (equal). **Mahmood Nikbakhtazadeh:** Validation (equal); Writing‐original draft (equal); Writing‐review & editing (equal). **Kourosh Azizi:** Conceptualization (lead); Formal analysis (lead); Funding acquisition (lead); Methodology (lead); Resources (lead); Supervision (lead); Validation (lead); Writing‐original draft (lead); Writing‐review & editing (lead).

### OPEN RESEARCH BADGES

This article has earned an Open Data Badge for making publicly available the digitally‐shareable data necessary to reproduce the reported results. The data is available at https://doi.org/10.5061/dryad.3bk3j9khv.

## Data Availability

All data have been made publicly available in Dryad with a DOI provided: https://doi.org/10.5061/dryad.3bk3j9khv.
